# Supplementary material for: BMP9-ID1 Signaling Activates HIF-1α and VEGFA Expression to Promote Tumor Angiogenesis in Hepatocellular Carcinoma
Source: Int J Mol Sci. 2022 Jan 27;23(3):1475. doi: 10.3390/ijms23031475 (PMC8835914; doi:10.3390/ijms23031475)
Supplement: Supplementary file 1 [file ijms-23-01475-s001.zip › ijms-1557147-supplementary.pdf]

Supplementary Materials:

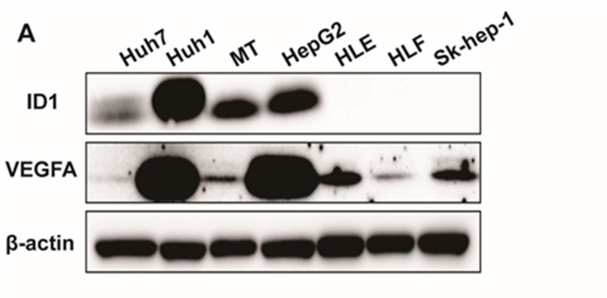

Figure S1. (A) Constitutive ID1 and VEGFA expression of representative HCC cell lines Huh7, Huh1, MT, HepG2, HLE, HLF, and Sk-hep-1.
